# Survival after Aluminum Phosphide Poisoning in Pregnancy

**DOI:** 10.1155/2020/2785425

**Published:** 2020-09-28

**Authors:** Adnane Lahlou, Mohammed Sidayne, Saïd Benlamkaddem, Mohamed Adnane Berdaï, Mustapha Harandou

**Affiliations:** Obstetric and Pediatric Intensive Care Unit, Hassan II Academic Hospital, Fez, Morocco

## Abstract

Intoxication and drug overdose as suicidal attempt are rare in pregnancy. We report here the case of aluminum phosphide poisoning in a pregnant lady through oral and intravaginal administration which was managed with aggressive supportive measures without resorting to extracorporeal life support.

## 1. Case Report

We report the case of a 35-year-old female who was admitted to our intensive care unit for aluminum phosphide (AlP) poisoning. She was found unresponsive beside empty AlP vials in her home before being brought to our ICU.

Upon admission in the ED (the exact time of the ingestion is unknown), she was unconscious with a Glasgow Coma Scale of 7 (eye opening 1/4, verbal response 2/5, and motor response 4/6), hypothermic with central temperature of 33°C, and had mottled and cold extremities. She was hypotensive with a blood pressure of 70/40 mmHg, with a heart rate of 50 beats per minute, and had shallow breathing with SpO_2_ at 60% on 10 liters on rebreathing mask. The remainder of the physical examination was remarkable for a distended abdomen with a fundal height above the umbilicus. A bedside point-of-care ultrasound was performed, showing global hypokinesia with an EF of 20%, a collapsed inferior vena cava (IVC), and at the pelvis, the fetus was with negative heart activity.

Resuscitation was commenced according to our department's protocol for the management of AlP poisoning [1]. Airway was secured with an endotracheal tube, gastric lavage with sodium bicarbonate solution, magnesium sulfate infusion, volume expansion with 250 cc of Ringer's lactate (RL) solution as well as dobutamine drip (7mcg/kg/min) on a central line (as per our local protocol, a dobutamine infusion is started early in aluminum phosphide poisonings with hemodynamic instability until a more advanced proper hemodynamic assessment is made [1]).

**Figure 1:**
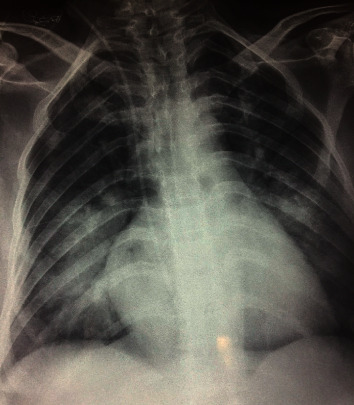
Admission Chest X-ray showing bilateral infiltrates.

**Figure 2:**
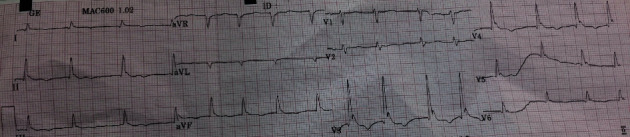
Admission EKG.

Bedside chest X-ray (Figure 1), EKG (Figure 2), toxicological, and full blood workup were ordered.

Chest X-ray found bilateral infiltrates with a normal cardiac silhouette.

Blood results found abnormal renal function tests: urea: 0.58 g/l, creatinine: 18 mg/l, and high troponine TnIc: 6.71 ng/ml (Table 1).

Admission EKG showed diffuse negative T wave, sinus bradycardia, and Osborn waves consistent with hypothermia.

Admission ABG: pH: 7.01, HCO_3−_: 11 mmol/l, PaCo_2_: 22 mmHg, and PaO_2_: 32 mmHg with a PaO_2_/FiO_2_ ratio of 80.

Toxicologic tests found evidence of phosphine using the silver nitrate test on the gastric content.

On admission to our ICU, the patient was still hypothermic with a temperature of 35°C with an improved hemodynamic status on 15 mcg/kg/min dobutamine (BP: 94/52 mmHg). She remained, however, oliguric and hypoxic with SpO_2_ reaching only 80% on 100% FiO_2_.

We decided to withhold labor induction after consulting with the hospital obstetricians. A control beside echo showed a slightly improved contractility on dobutamine with an EF of 30%, an ITV of 14, and an IVC measured at 20 cm. We decided to add norepinephrine 0.3 mcg/kg/min with a furosemide infusion. A better hemodynamic status was obtained with a mean arterial pressure >65 mmHg and acceptable diuresis (0.6 cc/kg/h).

However, with persistent hypoxia despite hemodynamic improvement, we considered ARDS and ventilated the patient subsequently with a SpO_2_ reaching 90%.

Upon hemodynamic and respiratory improvement, we decided to induce labor with intravaginal misoprostol after consulting with the obstetricians on the third day after admission. A male fetus was vaginally delivered the following day. Weaning from catecholamines, mechanical ventilation was started, and the patient was extubated on the fifth day under both low norepinephrine and dobutamine. She was discharged two days later after proper psychiatric counseling and weaning from catecholamines. A week later, the patient came back for a post-ICU consult, she was conscious, eupneic, stable, and exhibiting no signs of neither cardiac nor respiratory failure. She was followed up with the hospital psychiatry ward which deemed she could be treated as an outpatient.

## 2. Discussion

AlP poisoning is common especially in underdeveloped countries. AlP is the main compound of a rodenticide (PHOSTOXIN©) used to protect crops. It is formulated as a dark-gray 3 g-tablet having the smell of decaying fish. Its toxicity stems from the liberation of phosphine gas (PH_3_), produced upon contact with atmosphere or the gastric hydrochloric acid [2]. Phosphine enters immediately into the bloodstream. As to its toxicokinetics, animal studies on rats suggest an LC50 of 11 ppm (15 mg/m^3^) [3]. The gas can cause direct alveolar injury and is absorbed through the digestive tract and causes damage to the other organs by the inhibition of mitochondrial cellular respiration, cytochrome C oxidase inhibition, and subsequent production of free radicals [4]. The resulting pathophysiological effects of these cellular derangements are toxic myocarditis with severely decreased cardiac output and congestive heart failure and circulatory failure owing to a state of vasoplegia because of the effects on the vascular tissue, respiratory failure due to direct alveolar damage or the systemic effects of phosphine, as well as acute kidney injury related to direct injury or secondary hypoperfusion [5]. When the first reported cases were described, inhalation was the most common exposure route, whereas nowadays, it is ingested mostly as suicidal attempt especially in underdeveloped countries, several cases being reported from India, Iran and Morocco [6]. Intoxication in developed countries with AlP is very rare and reported as accidental [7]. In our case, the parturient had ingested an unknown quantity and cervix examination found an Alp pellet.

As there is no specific antidote for Alp, the treatment is supportive and consists on thorough decontamination, hemodynamic, and respiratory support. Following ingestion, gastric lavage, following airway protection when deemed necessary, should be performed with bicarbonate and potassium permanganate KMnO_4_(1 : 10000), and the latter oxidizes phosphine to nontoxic phosphate [5]. However, the first, though controversial [8], is supposed to decrease gastric hydrochloric acid liberation which enhances further phosphine liberation [9]. Some authors also suggested the use of coconut oil for lavage as it reduces the amount of absorbed phosphine [10]. Another novel and original approach to treatment was gastric ventilation to reduce phosphine production [11]. Magnesium sulfate should be administered as early as possible due to its antiarrythmic and its antiperoxidant effects; there is, however, no consensus on the regimen, authors suggesting an initial bolus of 3 g followed by an intermittent 1 g/6h infusion [11]. Poor hemodynamic status should be tackled aggressively, and dobutamine for inotropic support and norepinephrine for the resulting vasopelgia should be the mainstay of the first-line treatment. Should hypotension be refractory, authors suggested recourse to high euglycemic insulin therapy [12], due to the inotropic effects of insulin which have also shown to be effective in other states of refractory hypotension following drug overdose [13]. Other authors suggested the use of intralipid emulsion to neutralize the dissolved circulating phosphine [14]. Moreover, in cases of severe hemodynamic collapse not responding to usual or novel approaches, recourse to extracorporeal membrane oxygenation (ECMO) or intra-aortic balloon pump is increasingly reported [15, 16].

Our patient presented with multiple organ failure: respiratory, hemodynamic, neurologic, and renal failure. Aggressive and early management were paramount in the evolution. There is yet no antidote for aluminum phosphide [17]. As for the prognosis, Farzaneh et al. proposed a nomogram including admission GCS, systolic blood pressure, bicarbonate level, and urinary output to predict mortality [18].

## 3. Conclusion

Alp poisoning remains fatal and common in underdeveloped countries. The keystone of recovery is early supportive hemodynamic and respiratory management, which should be tailored to every patient and to the equipment and therapies available in each institution.

## Figures and Tables

**Table 1:** ABG and biological values evolution throughout the stay.

Value	Normal range	Admission	Two hours after admission	Day 2	Day 7 (discharge)
pH	7.38–7.42	7.01	7.12	7.25	7.34
HCO_3_^−^	22–26	11	13	17	23
PaO_2_	>75 mmHg	32	70	72	62
PaCO_2_	35–45 mmHg	22	25	35	32
PaO_2_/FiO_2_ ratio	>300	80	140	144	310
Troponin I-C	<0.04 ng/ml	6.71	8.02	5.04	0.06
Urea	0.1–0.4 g/l	0.58	0.63	0.87	0.43
Creatinine	7–14 mg/l	18	19	19	15
